# Massive Fatal Pulmonary Embolism While on Therapeutic Heparin Drip

**DOI:** 10.1177/2324709620914787

**Published:** 2020-03-25

**Authors:** Said Hajouli

**Affiliations:** 1 Hospital Medicine Department, Logan Regional Medical Center, Logan, WV, USA

**Keywords:** pulmonary embolism, venous thrombosis, heparin drip, anticoagulation, fatal PE, massive PE

## Abstract

Venous thromboembolism (VTE) includes deep venous thrombosis (DVT) and pulmonary embolism (PE). In this article, we present a case of a patient with an acute DVT who was treated with a therapeutic heparin drip, then developed syncope while in the hospital and found to have massive bilateral PEs. This case aims to arouse the medical staff’s awareness of the VTE diagnosis even if the patient is fully anticoagulated. We review the indications for DVT hospitalization, heparin infusion monitoring, risk factors for developing PE from DVT, mechanisms of developing PE from DVT while on therapeutic anticoagulation, and signs and treatment of massive PE.

## Introduction

Venous thromboembolism (VTE) is an important cause of cardiovascular disability and death. The yearly incidence of VTE in the United States is 1 to 2 per 1000.^1^ It affects 300 000 to 600 000 individuals in the United States every year.^1^ The mortality rate at 28 days, after the first VTE diagnosis, is 9.4% in deep venous thrombosis (DVT) and 15.1% in pulmonary embolism PE.^2^ PE is a major source of morbidity and mortality that causes 300 000 deaths annually in the United States.^3^ Massive PE is defined by obstruction of >50% of the cross-sectional area of the pulmonary arterial tree causing severe and acute right ventricular (RV) overload and cardiopulmonary failure. Seventy percent of patients who die of a PE die within the first hour of symptoms onset.^4^ Anticoagulation is the main treatment for VTE. Anticoagulation and thrombolysis are the standard treatments for acute massive PE.

## Case Presentation

**Figure 1.**
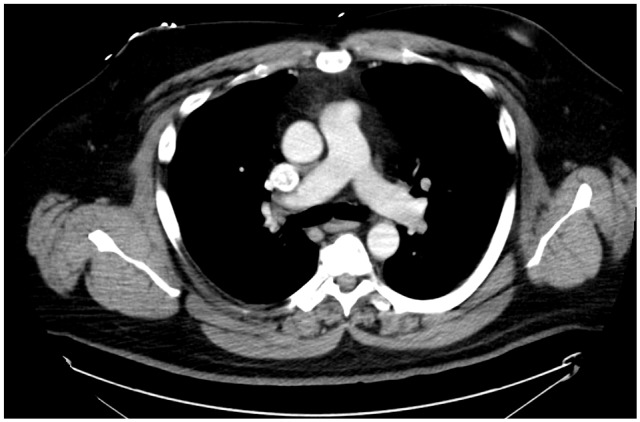
Computed tomography scan of chest with contrast. No pulmonary embolism.

**Figure 2.**
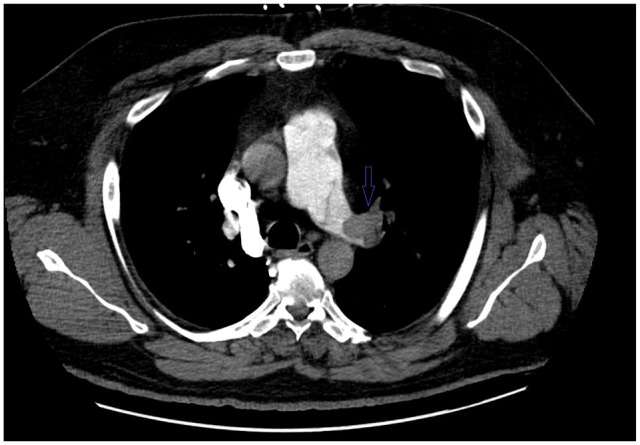
Computed tomography pulmonary embolism (CTPE) showing left pulmonary artery PE.

**Figure 3.**
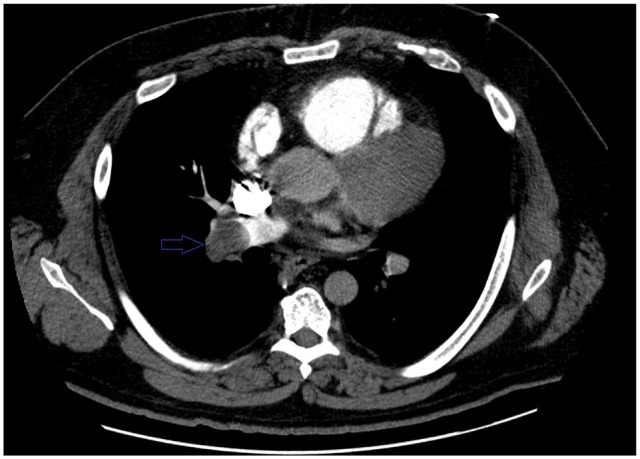
Computed tomography pulmonary embolism (CTPE) showing right pulmonary artery PE.

**Figure 4.**
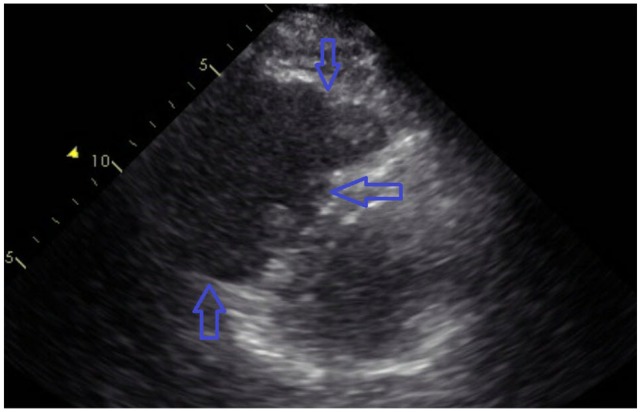
Echocardiogram showing right ventricular dilatation and hypokinesis.

**Figure 5.**
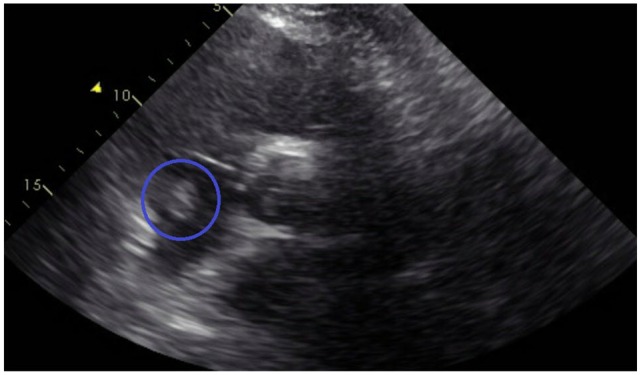
Echocardiogram showing thrombus in the right atrium.

A 46-year-old Caucasian male with a history of hypertension, obesity, hyperlipidemia, and left renal cell adenocarcinoma (RCC) a few years ago status post partial nephrectomy and was in remission, presented to the emergency room (ER) with a 10-day history of severe right lower extremity (LE) pain, redness, and swelling. No history of recent travel, trauma, immobilization, or surgery. No history of DVT in the past. He was not compliant with his medications that included Lisinopril and atorvastatin. His physical examination showed swelling of the right LE with erythema, edema, tenderness, and positive Homans’ sign. Dorsalis pedis and posterior tibial arteries pulses were +3 bilaterally. No cyanosis or blanching of the lower extremities. The rest of the physical examination was unremarkable. His basic laboratory workup including complete blood count, prothrombin time, partial thromboplastin time (PTT), international normalized ratio (INR), and the comprehensive metabolic panel was normal (Table 1). His LE venous Doppler showed acute DVT from the proximal right superficial femoral vein through the popliteal vein and involving the calf veins. The patient was given analgesics orally, but his pain did not subside so he was started on intravenous (IV) analgesics. The patient was admitted to the telemetry floor after he was started on a heparin drip with a bolus for full anticoagulation. Hypercoagulable state workups were sent. With his history of RCC, the patient had a computed tomography (CT) of the chest and abdomen/pelvis with IV contrast to rule out any masses/cancers as an underlying possible provoked cause of his acute DVT, but all came back negative. No incidental PE was seen in the CT chest (Figure 1). The patient was thought to have a high-risk DVT due to its extension, so the plan was to treat him with parenteral anticoagulation for 5 to 7 days and then switch to direct oral anticoagulation (DOAC). On day 5, the patient had an episode of syncope for 2 minutes when he was standing up from his bed. His vitals at that time were the following: blood pressure 121/67 mm Hg, heart rate 95 beats per minute, respiratory rate 16 breaths per minute, and oxygen saturation (SaO_2_) 96% on room air. Orthostatic vitals were negative. His electrocardiography (EKG) and telemetry did not show any arrhythmia. It was thought that the patient had a vasovagal reflex syncope. The patient was continued on heparin drip with an activated PTT (aPTT) 1.5 times the control all the time (Table 2). In a few minutes after his syncopal episode, the patient became hypoxic, tachypneic, and tachycardic. His repeated blood pressure was 128/75 mm Hg. His chest X-ray showed clear lungs. His repeated EKG showed sinus tachycardia with a heart rate of 101 beats per minute. His Wells score was 10.5, which is high risk for PE, so STAT CT-PE was done and showed bilateral large pulmonary emboli in both main pulmonary arteries extending into the upper and lower pulmonary arteries bilaterally with right ventricle appearing more prominent than the left suggesting right heart strain (Figures 2 and 3). STAT bedside echocardiogram showed severely enlarged RV with severely reduced RV systolic function and a large mobile mass in the right atrium (RA) suspected of a thrombus (Figures 4 and 5). A few minutes later, he was unresponsive and did not have a pulse. Advanced Cardiac Life Support (ACLS) was started per protocol for pulseless electrical activity (PEA) arrest. Tissue plasminogen activator infusion was administered per protocol. One hundred minutes after the ACLS, the patient remained pulseless and could not achieve a return of spontaneous circulation (ROSC) so the code was called out and the patient was pronounced dead. His hypercoagulable workup showed high factor II DNA analysis consistent with a single G20210A mutation (heterozygote) in factor II (prothrombin) gene. Factor V Leiden DNA was measured by allele-specific polymerase chain reaction and was high, which was consistent with a single R506Q mutation (heterozygote) in factor V gene.

**Table 1.** Laboratory results on admission.

WBC	10.4
Hemoglobin	15.9
Hematocrit	48.2
Platelet	294
INR	1
PT	10.4
PTT	26.8

Abbreviations: WBC, white blood cell; INR, international normalized ratio; PT, prothrombin time; PTT, partial thromboplastin time.

**Table 2.** aPTT monitoring.

Day of Hospitalization	aPTT Level (Normal Range 24.5-31.5)		
1 (every 6 hours)	59.5	49.5	50.6
2	72		
3	49.8		
4	50.4		
5	50.7		

Abbreviation: aPTT, activated partial thromboplastin time.

## Discussion

Venous thromboembolism is the third most common cardiovascular cause of death in the United States after heart attacks and strokes.^5^ It can be treated in an inpatient or in an outpatient setting. PE is usually treated in an inpatient setting with an average hospital stay of 6 days.^6^ The safety of treating PE at home is uncertain as acute PE is associated with higher short-term mortality than acute DVT. Because of limited evidence, the American College of Chest Physicians (ACCP) gives a grade 2B recommendation as for early discharge of low-risk PE patients if PE Severity Index (PESI score) <85, and none of any of the following: hypoxia, hypotension (systolic blood pressure <100 mm Hg), intense symptoms, thrombocytopenia with platelet count <70 000/mm^3^, recent bleeding, PE even with anticoagulation, or profound renal or liver dysfunction.^7^

Outpatient treatment of LEDVT does not increase the risk of complications or mortality when compared with inpatient treatment. There is a grade 1B recommendation from the 2012 ACCP guidelines for outpatient treatment of LEDVT whose home circumstances are adequate.^7^ However, there are some criteria that help determine which patient should be treated as inpatient, and the presence of one of them indicates the possible need for hospitalization^8^:

1. *Massive DVT*: 5% of symptomatic LEDVT can progress into the iliofemoral veins or even into the inferior vena cava and cause massive DVT. Massive DVT will lead to swelling of the whole leg, severe symptoms, acrocyanosis, and acute limb ischemia if not treated appropriately. Hospitalization of these patients is recommended for parenteral analgesia and to consider thrombolytic agents, or for extended duration of parenteral anticoagulation (like unfractionated heparin [UFH] or low-molecular-weight heparin [LMWH] for 10-14 days).
2. *High risk of bleeding*: 5% to 10% of newly diagnosed DVT patients are at high risk of bleeding when they start anticoagulation therapy and they should be monitored closely.^2^ Those include recent (within 1 week) surgery or trauma, thrombocytopenia (platelet count <100 × 10^6^/L), coagulopathy, bleeding within the past 4 weeks (like from a peptic ulcer), active bleeding, or advanced cancer with intracerebral or intrahepatic metastases as metastases to these sites are usually highly vascular.
3. *Concurrent symptomatic PE*: It is estimated that 10% of symptomatic LEDVT patients have also symptomatic PE at the time of DVT diagnosis and those patients require hospitalization.^9^
4. Inpatient treatment for acute LEDVT is also recommended for patients with severe pain that requires IV analgesics or patients with severe comorbidities (eg, advanced malignancies). Patients whose home environment is not appropriate are also treated as inpatients such as no phone access, no strong family support, unable to return to the hospital quickly if worsening, or any issues that impede the outpatient follow-ups and anticoagulation monitoring (such as psychological, cognitive, or physical impairments).

This patient was hospitalized as he required IV analgesics, was not compliant, and thought to have a high-risk DVT even he did not have a massive DVT.

The primary goal of VTE treatment is to reduce morbidity, mortality, clot propagation, and prevent recurrent thrombosis. Anticoagulation is the cornerstone of VTE treatment. DOAC is the preferred treatment for non–high-risk VTE.^10^ Parenteral anticoagulants like LMWH or UFH are recommended in the initial acute phase of high-risk VTE (first 7 days in which the inflammatory process is at its highest) and continued for at least 5 days.^7,11^ Heparin (continuous infusion or subcutaneous) was the standard of care for VTE treatment (grade 1A) until LMWH production. Heparin has multiple advantages such as short-acting time, inexpensive cost, rapid onset, and reversibility.^12^ It is the preferred treatment for hemodynamically unstable PE or iliofemoral DVT as those patients may require advanced therapies and interruption of their anticoagulation. Heparin interacts with antithrombin III and inhibits thrombin formation that leads to the prevention of fibrinogen conversion to fibrin, which prevents thrombosis. There is no optimal approach to monitor heparin therapy. The 2 available methods to monitor heparin are anti-Xa and aPTT. The aPTT is less expensive, more familiar to clinicians, and more available when compared with anti-Xa, and it is the most used method by medical providers to monitor heparin therapy. However, there are pre-analytic and analytic problems that may occur and may affect both aPTT and anti-Xa results such as inappropriate sample collection, transport, processing, storage, reagents used, and coagulometer used. There are also biological factors that may affect the aPTT but have less impact on the anti-Xa levels such as high levels of factor VIII or elevated fibrinogen.^13-16^ Anti-Xa is inaccurate in the setting of elevated total bilirubin levels to >6.6 mg/dL, elevated triglycerides to >360 mg/dL, and recent use of LMWH or fondaparinux (especially with renal dysfunction).^17,18^ Anti-Xa level does not require frequent monitoring tests or dose adjustment compared with aPTT.^18^ There are no sizeable VTE studies that looked into the outcomes of patients monitored with anti-Xa while on heparin treatment. The use of anti-Xa to monitor heparin therapy is recommended in the case of heparin resistance or prolonged baseline aPTT.^19^ The risks of recurrent VTE and thrombus extension were lower when aPTT was preserved between 1.5 and 2.5 times the control.^20,21^ These data produced the guidelines recommendations to keep the aPTT levels between 1.5 and 2.5 times the control as a therapeutic range.

Anticoagulation does not dissolve an existing thrombus but stabilizes it, prevents its extension, and decreases (but does not eliminate) the risk of embolization and recurrent thrombosis. Forty percent of symptomatic DVT patients have silent PE at the time of diagnosis.^22^ Our patient did not have PE at the time of his DVT diagnosis according to his first CT chest with IV contrast. There are no specific comorbidities or VTE risk factors that can increase the likelihood of developing PE in DVT patients. In a study of 141 PE patients,^23^ the overall prevalence rate of concomitant DVT was 45.4% and 39% had proximal DVT. There were no statistically significant differences between the 2 groups (PE without DVT vs PE with concomitant DVT) in terms of VTE risk factors and comorbidities. In another study that had 114 patients with DVT,^24^ the prevalence of PE was 52.6%. They divided patients into 2 groups and compared the risk factor for each group: group I had DVT patients without PE and group II had DVT patients with PE.^24^ The only risk factor that reached statistical significance in this study was infection (more in group II, with a *P* value of .005), and they concluded that infection was linked to an increased risk of PE development in patients with LEDVT.^25^ However, this study had a small sample size of 114 patients only. Acute infection is a known factor to increase the risk of PE and DVT.^26^

Pulmonary embolism while on therapeutic anticoagulation is uncommon and possesses a challenge diagnosis and management.^24^ The 2 possible mechanisms in which the PE may occur even with therapeutic anticoagulation are clot propagation (emboli break off from an existing DVT to the blood circulation and then to the pulmonary arteries) or breakthrough PE (new thrombus formation in the pulmonary system).

The occurrence of PE as an extension of DVT is infrequent in patients treated well with anticoagulation. The topical propagation of DVT is common and could be part of the natural history of VTE as a prolonged inflammatory and remodeling process. In randomized controlled trials that included serial screening imaging studies (venous duplex or venography) after DVT diagnosis, the risk of asymptomatic topical DVT propagation in the first 10 days of therapy was 4.7% and 1.1% in UFH and LMWH, respectively,^27^ and these may reach up to 30% to 38% in some other studies (most of these cases had subtherapeutic anticoagulation).^28,29^ The risk of symptomatic topical DVT propagation is 0.3% and 0.6% for UFH and LMWH, respectively.^28^ The data for asymptomatic and symptomatic PE propagation are less known, and the reliable estimates of developing fatal PE from acute DVT are nonexistent. In one study that had patients presented with DVT and were treated with heparin for 5 to 10 days and then oral anticoagulation, the risk of fatal PE was low at 0.4%.^30^

The risk of the breakthrough event, when anticoagulation is managed well, is 2 per 100 patient-years for oral anticoagulation,^31,32^ and it may warrant cancer screening as a possible underlying cause. The breakthrough rate while on warfarin was 1.2% in Kaiser Performance ER patients in a study that excluded patients with VTE in the previous 30 days,^33^ 31% had active cancer and 42% had at least one subtherapeutic INR 14 days before the ER visit. The incidence was 6.1% with therapeutic INR in an Australian study in 2013.^34^ This risk was 4% in the first 15 days of treatment with UHF or LMWH.^35,36^ In a prospective study that had 50 VTE patients (DVT, PE, or inferior vena cava thrombosis) treated with heparin drip and followed with repeated imaging at day 15, PE occurred in 2 patients (4%), one of them had subtherapeutic anticoagulation and died (2%).^36^ Fatal PE rate was 0.07% during treatment with warfarin or DOAC.^32^

The most common causes of the breakthrough PE are subtherapeutic anticoagulation (incorrect dose, drug interaction, or poor adherence) or underlying disease that can cause hypercoagulability such as active cancer,^37^ vasculitis (Behcet disease),^38^ myeloproliferative neoplasms,^39^ heparin-induced thrombocytopenia,^40^ vascular malformation,^41^ antiphospholipid antibody syndrome,^42^ and JAK2 V617F mutation.^31,43^

In our patient, inadequate anticoagulation was less likely as his aPTT was 1.5 times the control all the time (Table 2), which should significantly decrease the risk of recurrent thrombosis and prevent extension.^20,21^ Heparin resistance was also less likely as the patient did not require high doses of heparin to keep his aPTT at goal. Heparin-induced thrombocytopenia was excluded because of normal platelets 1 hour before the PEA arrest. His anticardiolipin antibodies and anti-B2-GPI antibodies were negative, which should make antiphospholipid antibody syndrome less likely too. He had a remote history of RCC but did not have active cancer at the time of presentation, and his CT scan of abdomen/pelvis did not show any masses. He did not have any signs or symptoms of vasculitis. His BCR-ABL and JAk2 mutations were negative, and his laboratory workup did not show increased red blood cell volume or thrombocytosis, which should rule out myeloproliferative neoplasms. The patient had both single G20210A mutation in the factor II gene and a single R506Q mutation in factor V gene, which increases the risk of recurrent thrombosis but the interval from the first DVT to recurrence is few years, not few days.^44^ In his case, we think that heparin failed to stabilize the thrombus in his LE and caused thrombus to break off from his LE into the bloodstream to RA and pulmonary system to cause massive fatal PE, which is not very common and would not be expected while on anticoagulation.

Symptoms of PE are nonspecific, and it can be difficult to diagnosis PE and even more difficult in patients on therapeutic anticoagulation. Syncope can be the presenting symptom of PE in 0.06% to 0.55% ER patients and 0.15% to 2.1% in hospitalized syncope patients,^45^ which may reach up to 6%.^46^ Massive PE is characterized by systemic hypotension and cardiogenic shock. Systemic hypotension is described as a systolic arterial pressure <90 mm Hg or a drop in systolic arterial pressure of at least 40 mm Hg for at least 15 minutes.^47^ Shock is defined by tissue hypoperfusion that leads to encephalopathy, decreased urine output, hypoxia, or cool and clammy limbs. Massive PE may be a challenging diagnosis if it arises in patients who have not been sick lately. The physical examination will show systemic hypotension, altered mental status, tachycardia, tachypnea, cyanosis, jugular vein distension, a parasternal heave, a tricuspid valve regurgitation murmur, and an accentuated P2. EKG may show sinus tachycardia, S1Q3T3 pattern, or T-wave inversions in V1 to V4, but ECG may be completely normal. Bedside echocardiogram must be done when a massive PE is suspected to confirm the RV dysfunction/dilatation and exclude other etiologies that can cause the same symptoms such as cardiac tamponade. Contrast-enhanced chest CT angiography is the gold standard for the diagnosis of PE.^48^ Fibrinolysis is the treatment of choice for the massive PE if no contraindication.^11,49,50^ It rapidly lyses the thrombus and improves the RV functioning. Treatment also includes volume expansion, vasopressors, and inotropes if needed. Massive PE may cause acute occlusion of the RV outflow that can lead to acute circulatory collapse and death. Most death takes place within a few hours after the onset of symptoms. The mortality rate in massive PE is 15.2% if arterial hypotension and 24.5% if cardiogenic shock.^47^ If PE causes cardiac arrest, the mortality rate will be high and thrombolysis should be attempted to achieve ROSC and probably better outcomes. However, even with thrombolysis in cardiac arrest related PE, the mortality rate is 90%.^51^

Other treatments of the massive PE if contraindications for thrombolysis exist include catheter thrombectomy and surgical embolectomy. The 2012 ACCP guidelines give grade 2C recommendations for catheter-assisted thrombus removal and surgical embolectomy and only if contraindications to thrombolysis exist and if surgical expertise and resources are available.^7^

The ACCP 2012 and 2016 guidelines recommend anticoagulation alone for the treatment of most acute DVTs. Other acute DVT treatments are thrombolysis (systemic or catheter-directed) and/or thrombectomy (surgical or catheter-directed), which are indicated only in patients with acute limb threat (phlegmasia cerulea dolens), massive iliofemoral DVT, or in which anticoagulation has failed. Those treatments are indicated only if patients have good functional status, low bleeding risk, fresh clot (<14 days old), and life expectancy >1 year.^11,12^ This is because the mortality rate and the risk of recurrent thrombosis are not changed with these interventions comparing with anticoagulation alone. Inferior vena cava filter is indicated only when anticoagulation is contraindicated (active bleeding).^52^ Our patient did not meet any of these criteria, and his acute DVT was treated according to the current guidelines with anticoagulation alone.

## Conclusion

Venous thromboembolism is still a possible diagnosis even if the patient is on full anticoagulation. PE is an important differential diagnosis of syncope. Keeping this in mind helps make an early diagnosis of a potentially fatal VTE and that may save patients’ lives. In this patient, his PE was diagnosed early and was treated with thrombolysis according to the current guidelines but he ended up in a PEA arrest and death, so more research may be required in the future to determine the risk factors for developing fatal PE from DVT while on anticoagulation and that can produce more guidelines to treat these patients and prevent their death.
